# Racial-Ethnic Disparities in Opioid Prescriptions at Emergency Department Visits for Conditions Commonly Associated with Prescription Drug Abuse

**DOI:** 10.1371/journal.pone.0159224

**Published:** 2016-08-08

**Authors:** Astha Singhal, Yu-Yu Tien, Renee Y. Hsia

**Affiliations:** 1 Health Policy and Health Services Research, Boston University School of Dental Medicine, Boston, Massachusetts, United States of America; 2 University of Iowa College of Pharmacy, Iowa City, Iowa, United States of America; 3 Department of Emergency Medicine and Philip R. Lee Institute for Health Policy Studies, University of California at San Francisco, San Francisco, California, United States of America; University of Stellenbosch, SOUTH AFRICA

## Abstract

Prescription drug abuse is a growing problem nationally. In an effort to curb this problem, emergency physicians might rely on subjective cues such as race-ethnicity, often unknowingly, when prescribing opioids for pain-related complaints, especially for conditions that are often associated with drug-seeking behavior. Previous studies that examined racial-ethnic disparities in opioid dispensing at emergency departments (EDs) did not differentiate between prescriptions at discharge and drug administration in the ED. We examined racial-ethnic disparities in opioid prescription at ED visits for pain-related complaints often associated with drug-seeking behavior and contrasted them with conditions objectively associated with pain. We hypothesized a priori that racial-ethnic disparities will be present among opioid prescriptions for conditions associated with non-medical use, but not for objective pain-related conditions. Using data from the National Hospital Ambulatory Medical Care Survey for 5 years (2007–2011), the odds of opioid prescription during ED visits made by non-elderly adults aged 18–65 for ‘non-definitive’ conditions (toothache, back pain and abdominal pain) or ‘definitive’ conditions (long-bone fracture and kidney stones) were modeled. Opioid prescription at discharge and opioid administration at the ED were the primary outcomes. We found significant racial-ethnic disparities, with non-Hispanic Blacks being less likely (adjusted odds ratio ranging from 0.56–0.67, p-value < 0.05) to receive opioid prescription at discharge during ED visits for back pain and abdominal pain, but not for toothache, fractures and kidney stones, compared to non-Hispanic whites after adjusting for other covariates. Differential prescription of opioids by race-ethnicity could lead to widening of existing disparities in health, and may have implications for disproportionate burden of opioid abuse among whites. The findings have important implications for medical provider education to include sensitization exercises towards their inherent biases, to enable them to consciously avoid these biases from defining their practice behavior.

## Introduction

Abuse of prescription opioids has been cited as the fastest growing drug problem in the United States [1], and has surpassed use of cocaine and heroin combined as the cause of mortality [2,3]. In 2013, nearly two million Americans abused prescription opioids and 16,235 deaths were attributed to prescription opioids representing almost a fourfold increase since 1999 [4,5,6]. In an attempt to curb this public health problem and reduce prescription of opioids to ‘drug-seekers’, numerous state and hospital opioid prescription guidelines have been developed and disseminated [7,8]. However, it is often impossible for physicians, especially emergency physicians who see any individual patient sporadically, to definitively determine whether a patient is a drug abuser, unless complete prior history is available through prescription drug monitoring programs [8].

On the other hand, inadequately treated pain in emergency departments (EDs) is also a major concern [9,10] when considering the recent decline in opioid prescription rates at EDs [11]. When patients present with pain-related complaints at the ED, it presents a quandary for the emergency physicians, who need to make decisions and react promptly. Hence, emergency physicians rely on several cues [12–14], often without being conscious of them, to subjectively determine whether or not to prescribe opioids for patients presenting with pain-related complaints.

Patients’ race-ethnicity is one such factor that may affect a physician’s decision to prescribe opioids [10,12]. Racial-ethnic disparities in opioid prescription have been documented nationally, with minorities being less likely to receive opioids [15–17]. However, previous studies did not differentiate whether opioids were administered in the ED or prescribed at discharge from the ED [15–19]. This is an important distinction when examining racial-ethnic disparities in opioid prescriptions for two reasons: 1) non-white race is one of the largest predictor of provider mistrust [12,13], and the aforementioned modes of dispensing opioids require very different levels of provider-patient trust; 2) while prescribing opioids contributes to the prescription opioid epidemic, one-time administration of opioid in ED does so minimally [18].

Certain conditions have long been associated with drug-seeking behavior, such as back pain and abdominal pain [20–23], partly because they often do not have visible clinical and/or diagnostic presentations [10]. Recent literature points to a consistent increase in ED visits for dental diseases [23–26], another common presentation among drug-seeking patients [21, 23]. These conditions, hereafter referred to as “non-definitive conditions”, are in sharp contrast to other pain-related conditions, such as long-bone fracture and kidney stones, that have more objective clinical presentation and can be confirmed with simple diagnostic tools. These conditions are referred to as “definitive conditions” for purposes of this paper.

We hypothesized that racial-ethnic disparities would be present in opioid prescription for non-definitive conditions (back pain, abdominal pain and toothache) that have been associated with drug-seeking behavior, but not among definitive conditions (long-bone fractures and kidney stones). Our secondary hypothesis was that racial-ethnic disparities would be more pronounced in opioids prescribed at discharge from the ED, as compared to opioids administered in the ED, as the former requires more provider-patient trust.

## Methods

### Data Source

We used the National Hospital Ambulatory Medical Care Survey (NHAMCS) data, which is based on a national sample of visits to emergency departments, as well as outpatient departments and ambulatory surgical centers, of non-federal general and short-stay hospitals [27]. We used NHAMCS data from five years, 2007 through 2011 (latest available), which is made available by the Centers for Disease Control and Prevention (CDC). Data prior to 2005 did not include information about whether medications were administered in the ED or prescribed at discharge, and data prior to 2007 did not include the ‘number of ED visits to same ED in past 12 months’ variable- which could have implications on ED physicians’ impression of whether a patient might be ‘drug-seeker’. De-identified, publically available data were used that were exempt from institutional review board review.

### Study Population

All ED visits made by adults aged 18–65 years for either a non-definitive or definitive condition during 2007–2011 were included. These conditions were defined using criteria from Pletcher et.al. study [15], as follows: Non-definitive conditions were defined using primary reason for visit (RFV) codes, include: (1) Toothache (15001); (2) Back Pain (19051); and (3) Abdominal Pain (15450, 15451, 15452, or 15453). Definitive conditions were defined using the primary diagnosis International Classification of Diseases 9th Revision-Clinical Modification (ICD-9-CM) codes, include: (1) Long-bone fractures (812, 813, 821, or 823); and (2) Kidney stones (592).

### Variable Definitions

The race-ethnicity variable was the key predictor, and was categorized into non-Hispanic white, non-Hispanic black, Hispanic and non-Hispanic other. One particular important covariate was severity of pain, with a rationale that the odds of prescribing opioids would be determined by pain severity. Pain severity was categorized into none (pain score = 0), mild/moderate (pain score = 1–6), severe (pain score = 7–10) and unknown (pain score = 99 or missing). Other covariates included age, sex, type of insurance, location of the ED, whether the ED visit occurred on a weekend, and number of times the patient had visited same ED in past 12 months. The year of ED visit was included in the model to examine any secular trends. We also examined the presence of drug-dependence indicator [(RFV = 11450, 11500, 45181, 23200, 23210, 45180, 45181, 45182, 59100, 59150), or (ICD9-CM = 290, 291, 292, 303, 304, 305.0, 305.5, 305.6, 648.3)].

Up to 8 medications were recorded for each ED visit, that we classified into opioid and non-opioid analgesics using the MULTUM Lexicon^®^ Drug Database Classification System [28]. Opioids included narcotic analgesics or narcotic analgesic combinations (Level 3 Lexicon code: 060,191). Non-opioids included nonsteroidal anti-inflammatory agents, salicylates, analgesic combinations, anti-migraine agents, and COX-2 inhibitors. Each medication had a corresponding variable indicating whether it was prescribed at discharge, administered in the ED or both. Two primary outcome variables were created to indicate whether a patient received an opioid prescription at discharge from the ED and whether opioid was administered in the ED. If a patient received opioid at both times, at ED and at discharge, they were counted in both outcomes.

### Analytic Methodology

To account for the complex survey design, weights, strata and primary sampling units provided with NHAMCS data were applied in all analyses. Descriptive analyses were conducted to examine the sample distribution across all covariates for each of the non-definitive and definitive conditions. Rates of opioid administration in the ED and prescription at discharge were also examined. Multivariate logistic regressions were used to assess the racial-ethnic disparities in both modes of opioid dispensing. In the first model, we assessed the racial-ethnic differences in odds of opioid administration and prescription after adjusting for pain severity. In the second full model, we controlled for all covariates described above. These multivariate regressions were conducted separately for opioid administration and prescription for each of the five conditions. All analyses were conducted using SAS version 9.4. Statistical significance was determined at p<0.05 using 2-tailed tests and 95% confidence intervals were used as precision estimates.

## Results

During 2007–2011, an estimated 36.5 million ED visits were made nationally by non-elderly adults for abdominal pain, followed by 14.3 million visits for back pain, 6.9 million visits for toothache, 3.4 million visits with primary diagnosis of kidney stones, and 2.1 million visits for long-bone fractures. Descriptive results from Table 1 revealed that greater proportion of ED visits for non-definitive conditions (toothache, abdominal pain and back pain) were made by racial-ethnic minorities and younger adults compared to ED visits for definitive conditions (long-bone fractures and kidney stones). About 27–47% of ED visits were made by patients with private insurance, except for toothache, where the majority of ED visits were made by either the uninsured or Medicaid beneficiaries. Compared to patients with definitive conditions, a greater proportion of patients with non-definitive conditions had repeatedly visited the ED in the past year. The majority of ED visits were associated with severe pain for all included conditions.

**Table 1:** Characteristics of ED Visits by non-elderly adults for selected pain-related reasons/diagnoses in US, 2007–2011.

		NON-DEFINITIVE CONDITIONS			DEFINITIVE CONDITIONS	
		Toothache	Abdominal Pain	Back Pain	Long-Bone Fracture	Kidney Stones
		%*	%*	%*	%*	%*
**Sample N**		1756	9497	3691	594	890
**Weighted N**		6875481	36510693	14333976	2103599	3416284
Race Ethnicity						
	Non-Hispanic White	62.7	57.4	64.5	71.7	79.0
	Non-Hispanic Black	25.9	23.5	22.6	14.5	6.2
	Hispanic	8.8	15.5	10.5	11.5	12.1
	Non-Hispanic Other	2.6	3.6	2.4	2.4	2.6
Age Category						
	18–35 years	66.1	53.3	42.8	27.0	38.9
	36–45 years	18.4	19.6	25.7	19.4	28.0
	46–65 years	15.6	27.2	31.5	53.7	33.2
Sex						
	Males	44.9	30.2	46.3	44.6	59.3
	Females	55.1	69.8	53.8	55.4	40.7
Insurance Type						
	Private Insurance	14.5	31.2	27.4	34.7	46.8
	Medicare	5.3	5.8	7.7	6.0	3.0
	Medicaid	24.5	19.2	16.5	10.5	7.8
	Uninsured	27.6	16.8	18.7	16.9	13.6
	Other/Unknown	28.0	26.9	29.8	31.9	28.9
Location						
	Non-Metropolitan Area	18.8	13.1	16.2	17.8	17.6
	Metropolitan Area	81.2	86.9	83.8	82.2	82.4
Weekend						
	No	70.2	72.9	72.1	62.4	70.8
	Yes	29.8	27.1	27.9	37.6	29.2
Repeated ED visits in past year						
	None/ Unknown	60.6	65.8	63.5	74.5	71.1
	Less than 2	22.5	21.3	21.7	18.1	22.2
	3 or more	16.9	12.9	14.9	7.4	6.7
Pain Severity						
	None	0.7	2.6	0.9	2.4	1.3
	Mild	2.5	6.6	4.4	4.5	4.8
	Moderate	16.7	22.4	19.6	19.6	13.8
	Severe	67.2	54.3	61.1	61.0	69.2
	Unknown	13.0	14.2	14.0	12.6	10.9
Year						
	2007	17.9	17.5	18.3	19.8	21.3
	2008	18.7	17.5	19.1	20.8	18.2
	2009	20.2	19.9	20.8	20.0	21.0
	2010	21.5	21.8	19.9	17.3	19.3
	2011	21.7	23.2	21.9	22.1	20.2

*Percentages may add up to more than 100 due to rounding up.

A majority of the ED visits for all conditions included in the study led to patients receiving either opioids or non-opioid analgesics, ranging from 61% for abdominal pain to 92% for kidney stones (Table 2). Also, a majority received an opioid that was either administered at ED or prescribed at discharge, with a greater proportion of ED visits for definitive conditions receiving opioids (80–85%) than non-definitive conditions (52–65%). Among the five conditions, ED visits for toothache were the least likely (30%) to have opioids administered in the ED, but 57% of them resulted in an opioid prescription at discharge. On the other hand, ED visits for abdominal pain were the least likely (25%) to result in an opioid prescription at discharge, although 44% of them led to opioid administration in the ED. As the proportion with drug dependence was less than 0.5% for all conditions, we did not include it in our models.

**Table 2:** Non-elderly adults who received pain medication as a prescription at discharge from ED, or administered at the ED for selected pain-related reasons/diagnoses in US, 2007–2011.

	NON-DEFINITIVE CONDITIONS			DEFINITIVE CONDITIONS	
	Toothache, %*	Abdominal Pain, %*	Back Pain, %*	Long-Bone Fracture, %*	Kidney Stones, %*
**Received any pain reliever (opioids or non-opioids) either in ED or at discharge**	76.4	61.2	80.9	87.4	92.3
**Received opioid either in ED or at discharge**	64.9	52.3	52.3	80.2	84.8
**Received opioids in ED**	29.6	43.6	41.4	61.6	65.9
**Received opioids at discharge**	57.0	24.8	42.6	53.7	58.9
**Had drug dependence**	0.0	0.4	0.3	0.2	0.0

*Percentages do not add up as these are not mutually exclusive categories.

Table 3 shows the odds of receiving a prescription for opioids at discharge from ED and opioids administered in the ED by race-ethnicity, after adjusting for severity of pain. There were significant racial-ethnic disparities in both opioid prescription at discharge as well as administration at ED visits for back pain and abdominal pain, even after accounting for variation in pain severity. Non-Hispanic blacks had significantly lower odds (adjusted OR ranging from 0.51 to 0.67) of receiving opioids during their ED visits for back pain and abdominal pain compared to non-Hispanic whites. However, no racial-ethnic differences in opioid prescription/administration were found among ED visits for toothache, and the definitive conditions. Pain severity was a consistent predictor of receiving opioids for all conditions in both models, except for kidney stones.

**Table 3:** Odds of receiving opioid as prescription at discharge from ED, or administered at the ED for selected pain-related reasons/diagnoses by race-ethnicity adjusted for pain severity.

**Odds of getting opioid PRESCRIPTION at DISCHARGE from the ED by race-ethnicity after adjusting for pain severity**											
		**Toothache**		**Abdominal Pain**		**Back Pain**		**Long-Bone facture**		**Kidney Stone**	
		**OR (95% CI)**	**p-value**	**OR (95% CI)**	**p-value**	**OR (95% CI)**	**p-value**	**OR (95% CI)**	**p-value**	**OR (95% CI)**	**p-value**
**Race-ethnicity**			0.3620		**<0.0001**		**0.0130**		0.3008		0.3615
	Non-Hispanic White	1		1		1		1		1	
	Non-Hispanic Black	0.78 (0.59–1.04)		**0.57 (0.47–0.70)**		**0.67 (0.52–0.88)**		1.00 (0.53–1.89)		0.76 (0.40–1.47)	
	Hispanic	0.74 (0.44–1.24)		0.82 (0.66–1.01)		0.79 (0.60–1.03)		1.87 (0.98–3.56)		0.86 (0.50–1.46)	
	Non-Hispanic Other	0.79 (0.38–1.64)		**0.65 (0.48–0.90)**		0.70 (0.43–1.14)		0.88 (0.28–2.76)		1.86 (0.74–4.67)	
**Pain Severity**			**0.0006**		**<0.0001**		**<0.0001**		**0.0242**		0.5467
	None/ Unknown	1		1		1		1		1	
	Mild/Moderate	1.34 (0.84–2.14)		0.98 (0.77–1.25)		1.04 (0.78–1.39)		1.76 (0.95–3.26)		0.90 (0.51–1.57)	
	Severe	**2.15 (1.34–3.46)**		**1.51 (1.22–1.86)**		**1.71 (1.29–2.27)**		**2.31 (1.26–4.24)**		1.14 (0.69–1.89)	
**Odds of getting opioid ADMINISTERED at the ED by race-ethnicity after adjusting for pain severity**											
**Race-ethnicity**			0.7863		**<0.0001**		**<0.0001**		0.2827		0.8487
Non-Hispanic White		1		1		1		1		1	
Non-Hispanic Black		0.97 (0.70–1.35)		**0.51 (0.43–0.62)**		**0.59 (0.47–0.74)**		0.83 (0.44–1.59)		1.04 (0.54–2.01)	
Hispanic		1.25 (0.76–2.07)		0.85 (0.70–1.03)		0.97 (0.72–1.30)		0.60 (0.35–1.03)		0.87 (0.44–1.70)	
Non-Hispanic Other		0.87 (0.44–1.72)		1.03 (0.77–1.39)		1.05 (0.62–1.78)		1.26 (0.29–5.47)		1.32 (0.62–2.80)	
**Pain Severity**			**0.0010**		**<0.0001**		**<0.0001**		**0.0052**		**0.0005**
None/ Unknown		1		1		1		1		1	
Mild/Moderate		1.69 (0.91–3.16)		0.92 (0.75–1.13)		0.77 (0.54–1.10)		0.77 (0.42–1.44)		0.77 (0.39–1.49)	
Severe		**2.27 (1.45–3.57)**		**2.64 (2.18–3.19)**		**1.88 (1.43–2.48)**		1.68 (0.97–2.92)		**1.96 (1.11–3.48)**	

Bold Odds Ratios (OR) and 95% Confidence Intervals (95% CI) were significant at p<0.05

Results from the full logistic regression models modeling the odds of receiving an opioid prescription at discharge from ED and administration of opioids at ED are presented in Tables 4 and 5, respectively. For back pain, compared to non-Hispanic whites, non-Hispanic blacks had 0.67 and 0.58 times the odds of receiving an opioid prescription at discharge and opioid administration in the ED, respectively. Among ED visits for abdominal pain, all racial-ethnic minorities had significantly lower odds of receiving a prescription for opioids at discharge, whereas non-Hispanic blacks had half the odds of non-Hispanic whites to have opioids administered at ED. Again, no such racial-ethnic differences in opioid prescription/administration were found among ED visits for toothache, long-bone fracture, and kidney stones.

**Table 4:** Multivariable logistic regression modeling the odds of receiving opioid as prescription at discharge from the ED for selected pain-related reasons/diagnoses in US, 2007–2011.

		Toothache		Abdominal Pain		Back Pain		Long-Bone facture		Kidney Stone	
		Adj. OR (95% CI)	p-value	Adj. OR (95% CI)	p-value	Adj. OR (95% CI)	p-value	Adj. OR (95% CI)	p-value	Adj. OR (95% CI)	p-value
Race-ethnicity			0.5121		**<0.0001**		**0.0284**		0.2058		0.4624
	Non-Hispanic White	1		1		1		1		1	
	Non-Hispanic Black	0.82 (0.61–1.10)		**0.56 (0.46–0.68)**		**0.67 (0.51–0.89)**		0.79 (0.42–1.48)		0.81 (0.42–1.57)	
	Hispanic	0.75 (0.45–1.26)		**0.79 (0.64–0.98)**		0.79 (0.60–1.04)		1.81 (0.86–3.80)		0.83 (0.46–1.49)	
	Non-Hispanic Other	0.80 (0.39–1.62)		**0.63 (0.45–0.86)**		0.70 (0.42–1.15)		0.48 (0.14–1.64)		1.71 (0.70–4.19)	
Age Category			0.2497		0.0898		0.8696		**0.0341**		0.0745
	18–35 years	1		1		1		1		1	
	36–45 years	1.24 (0.95–1.62)		1.11 (0.94–1.31)		0.98 (0.79–1.23)		1.23 (0.68–2.25)		0.67 (0.42–1.06)	
	46–65 years	0.98 (0.70–1.36)		0.92 (0.79–1.08)		1.04 (0.86–1.26)		0.66 (0.39–1.12)		1.13 (0.72–1.77)	
Sex			0.6804		**0.0035**		0.3451		**0.0012**		0.2039
	Males	1		1		1		1		1	
	Females	0.95 (0.74–1.22)		**1.21 (1.06–1.37)**		0.93 (0.80–1.08)		**2.27 (1.38–3.72)**		0.82 (0.59–1.12)	
Insurance Type*			0.1933		0.1242		0.3966		0.0528		0.4868
	Private Insurance	1		1		1		1		1	
	Medicaid	0.75 (0.51–1.11)		**0.79 (0.64–0.98)**	** **	0.85 (0.64–1.12)		1.74 (0.73–4.16)		0.96 (0.48–1.92)	
	Uninsured	0.85 (0.58–1.25)		0.97 (0.79–1.19)		1.05 (78–1.42)		**2.49 (1.30–4.76)**		1.49 (0.84–2.64)	
	Other/Unknown	0.57 (0.33–0.97)		0.86 (0.71–1.05)		0.86 (0.66–1.13)		1.55 (0.82–2.96)		1.36 (0.74–2.49)	
Location			0.6311		**0.0062**		0.6627		**0.0395**		0.0832
	Non-Metropolitan Area	1		1		1		1		1	
	Metropolitan Area	1.08 (0.78–1.51)		**1.44 (1.11–1.87)**		1.11 (0.70–1.74)		**1.64 (1.02–2.64)**		1.46 (0.95–2.24)	
Weekend			**0.0015**		0.7509		**0.0376**		0.2612		0.4959
	No	1		1		1		1		1	
	Yes	**1.50 (1.17–1.94)**		1.02 (0.89–1.17)		**1.20 (1.01–1.42)**		1.29 (0.83–2.01)		1.17 (0.74–1.85)	
Repeated ED visits in past year			0.845		0.7999	** **	0.9892		**0.0292**		0.1491
	None/ Unknown	1		1		1		1		1	
	Less than 2	1.07 (0.77–1.48)		0.98 (0.80–1.18)		0.98 (0.78–1.23)		1.25 (0.79–1.98)		0.93 (0.60–1.45)	
	3 or more	1.11 (0.77–1.61)		0.93 (0.76–1.15)		1.00 (0.78–1.28)		**0.43 (0.21–0.88)**		0.48 (0.23–1.01)	
Pain Severity			**0.0006**		**<0.0001**		**<0.0001**		**0.0238**		0.283
	None/ Unknown	1		1	** **	1		1		1	
	Mild/ Moderate	1.34 (0.84–2.14)		0.99 (0.77–1.26)		1.02 (0.76–1.36)		**1.84 (1.03–3.28)**		0.85 (0.49–1.49)	
	Severe	**2.19 (1.36–3.50)**		**1.51 (1.21–1.88)**		**1.72 (1.30–2.27)**		**2.35 (1.27–4.34)**		1.19 (0.71–1.98)	
Year			0.3213		0.9543		0.2611		**0.0200**		0.7453
	2007	1		1		1		1		1	
	2008	0.71 (0.43–1.17)		1.03 (0.77–1.36)		0.98 (0.70–1.36)		1.43 (0.68–3.00)		0.67 (0.31–1.44)	
	2009	0.78 (0.51–1.21)		1.09 (0.86–1.39)		0.93 (0.68–1.28)		**2.21 (1.05–4.68)**		0.92 (0.42–2.04)	
	2010	0.64 (0.38–1.06)		1.08 (0.84–1.39)		0.93 (0.73–1.19)		**2.79 (1.33–5.85)**		0.83 (0.38–1.79)	
	2011	**0.56 (0.33–0.96)**		1.03 (0.79–1.34)		0.72 (0.50–1.03)		1.06 (0.51–2.18)		0.75 (0.36–1.54)	

*Due to small proportion of ED visits that were made by Medicare enrollees, they were combined with the ‘Other/Unknown’ insurance type

Bold Adjusted Odds Ratio (Adj. OR) and 95% Confidence Intervals (95% CI) were significant at p<0.05

**Table 5:** Multivariable logistic regression modeling the odds of opioids being administered at ED for selected pain-related reasons/diagnoses in US, 2007–2011.

		Toothache		Abdominal Pain		Back Pain		Long-Bone facture		Kidney Stone	
		Adj. OR (95% CI)	p-value	Adj. OR (95% CI)	p-value	Adj. OR (95% CI)	p-value	Adj. OR (95% CI)	p-value	Adj. OR (95% CI)	p-value
Race-ethnicity			0.7696		**<0.0001**		**<0.0001**		0.4495		0.8485
	Non-Hispanic White	1		1		1		1		1	
	Non-Hispanic Black	1.01 (0.73–1.41)		**0.50 (0.42–0.59)**		**0.58 (0.45–0.73)**		0.83 (0.42–1.64)		0.88 (0.47–1.64)	
	Hispanic	1.30 (0.76–2.32)		0.85 (0.70–1.04)		0.93 (0.69–1.25)		0.64 (0.35–1.17)		0.78 (0.42–1.45)	
	Non-Hispanic Other	0.88 (0.44–1.77)		0.98 (0.73–1.32)		1.07 (0.63–1.83)		1.52 (0.32–7.32)		1.10 (0.47–2.58)	
Age Category			0.7189		**<0.0001**		**0.0001**		0.3648		0.6074
	18–35 years	1		1		1	** **	1		1	
	36–45 years	1.08 (0.79–1.47)		**1.50 (1.31–1.72)**		1.06 (0.85–1.32)	** **	1.56 (0.84–2.89)		1.26 (0.77–2.08)	
	46–65 years	1.18 (0.77–1.80)		**1.36 (1.20–1.55)**		**1.51 (1.23–1.86)**	** **	1.11 (0.68–1.81)		1.25 (0.75–2.09)	
Sex			0.3675		0.4009		**0.0218**		0.5467		0.6314
	Males	1		1		1	** **	1		1	
	Females	1.14 (0.86–1.52)		0.95 (0.85–1.07)		**1.19 (1.03–1.39)**	** **	0.88 (0.58–1.34)		1.10 (0.76–1.58)	
Insurance Type*			0.0763		**0.0008**		**0.0159**		0.8516		0.6753
	Private Insurance	1		1		1		1		1	
	Medicaid	**0.61 (0.38–0.99)**		**0.79 (0.67–0.93)**	** **	0.83 (0.62–1.13)		0.75 (0.35–1.59)		0.64 (0.31–1.31)	
	Uninsured	0.80 (0.53–1.22)		**0.78 (0.65–0.93)**		**0.63 (0.47–0.84)**		0.82 (0.42–1.60)		0.95 (0.54–1.66)	
	Other/Unknown	0.52 (0.31–0.89)		1.03 (0.87–1.21)		0.81 (0.64–1.03)		0.85 (0.44–1.50)		0.99 (0.53–1.85)	
Location			0.8699		**0.0003**		0.1983		0.4968		**0.0015**
	Non-Metropolitan Area	1		1		1		1		1	
	Metropolitan Area	0.96 (0.59–1.56)		**1.95 (1.36–2.79)**		1.29 (0.88–1.90)		0.81 (0.43–1.50)		**2.72 (1.47–5.04)**	
Weekend			0.8985		0.9660		**0.0219**		**0.0189**		0.7047
	No	1		1		1		1	** **	1	
	Yes	1.02 (0.74–1.41)		1.00 (0.89–1.12)		**1.27 (1.04–1.55)**		**1.68 (1.09–2.58)**		1.07 (0.76–1.52)	
Repeated ED visits in past year			0.2168		**<0.0001**	** **	0.6009		**0.0019**		0.4192
	None/ Unknown	1		1		1		1		1	
	Less than 2	1.16 (0.82–1.63)		1.03 (0.88–1.20)		1.08 (0.88–1.33)		**0.52 (0.29–0.93)**		0.80 (0.51–1.27)	
	3 or more	1.42 (0.93–2.18)		**1.49 (1.24–1.79)**		1.16 (0.87–1.42)		**0.36 (0.18–0.75)**		1.21 (0.61–2.40)	
Pain Severity			**0.0028**		**<0.0001**		**<0.0001**		**0.0027**		**<0.0001**
	None/ Unknown	1		1	** **	1		1		1	
	Mild/ Moderate	1.63 (0.88–3.01)		0.93 (0.76–1.14)		0.75 (0.53–1.06)		0.75 (0.40–1.42)		0.76 (0.38–1.52)	
	Severe	**2.15 (1.36–3.39)**		**2.68 (2.24–3.21)**		**1.83 (1.40–2.40)**		**1.81 (1.00–3.28)**		**2.17 (1.19–3.96)**	
Year			0.9448		0.4247		0.2439		0.8205		0.4140
	2007	1		1		1		1		1	
	2008	1.01 (0.58–1.78)		1.19 (0.95–1.50)		1.30 (0.95–1.77)		1.04 (0.49–2.23)		1.78 (0.76–4.16)	
	2009	0.84 (0.47–1.51)		1.16 (0.90–1.50)		1.03 (0.78–1.36)		1.33 (0.56–3.14)		1.16 (0.47–2.84)	
	2010	0.91 (0.52–1.59)		1.22 (0.99–1.50)		1.16 (0.85–1.56)		1.44 (0.66–3.13)		1.27 (0.52–3.11)	
	2011	0.85 (0.45–1.60)		1.14 (0.91–1.42)		0.94 (0.67–1.31)		1.16 (0.45–2.95)		1.64 (0.74–3.65)	

*Due to small proportion of ED visits that were made by Medicare enrollees, they were combined with the ‘Other/Unknown’ insurance type.

Bold Adjusted Odds Ratio (Adj. OR) and 95% Confidence Intervals (95% CI) were significant at p<0.05

When examining other covariates associated with opioid prescription (Table 4), we found that ED visits that occurred on a weekend had a 50% and 20% greater odds of receiving an opioid for toothache and back pain respectively, compared to ED visits occurring on weekdays. Pain was a significant predictor, wherein patients with severe pain were more likely to receive opioid prescription for all conditions, except kidney stones.

Similarly, when examining covariates associated with opioid administration (Table 5), we found type of insurance, past ED visits, and pain severity to be significantly associated. Among non-definitive conditions, the odds of opioids being administered were 61% for Medicaid enrollees presenting with toothache, 63% for the uninsured with back pain, and 78% for Medicaid enrollees and uninsured, compared to the privately insured. Number of repeated ED visits in the past 12 months was significantly associated with opioid being administered for ED visits for abdominal pain and long-bone fracture. Pain severity was positively associated with opioid administration at the ED for all conditions.

## Discussion

To our knowledge, this is the first study to examine racial-ethnic disparities in opioid prescriptions, while distinguishing drug prescriptions from drug administration in the ED, in conditions commonly associated with drug-seeking behavior in contrast with objective painful diagnoses. In this analysis of nationally representative data of ED visits over five years, we found that racial-ethnic disparities exist in both, opioid prescription and administration, during ED visits for back pain and abdominal pain, but not during visits for toothache, kidney stones and long-bone fracture. Our hypothesis was partially confirmed as racial-ethnic disparities were persistent among ED visits for non-definitive conditions, except toothache, but not for definitive conditions, after adjusting for covariates.

Several studies have examined racial-ethnic disparities in opioid prescription, without distinguishing prescriptions given at discharge from opioid administration at the ED [15–19]. However, prescribing opioids at discharge grants the patient greater access to opioids, hence requiring more trust from the physicians. Moreover, prescribing opioids has the potential to contribute to the prescription drug overdoses, while administration of opioids in the ED does not [18]. Current study distinguishes between prescription at discharge and administration in ED and found that racial-disparities exist in both types of opioid dispensing.

The evidence behind differential racial disparities based on types of diagnoses and conditions is conflicting. Many studies report no racial-ethnic disparities in opioid prescriptions for ED visits for long-bone fracture [16,29], yet others report significant racial-ethnic disparities for long-bone fractures and kidney stones [15,30]. Our findings are similar to a study that shows significant racial-ethnic disparities in opioid prescription for less-objective conditions such as migraine or back pain, but no such disparities for long-bone fractures [16]. However, that study did not differentiate opioids that were prescribed at discharge from ED from those that were administered in the ED. We were able to make this differentiation and our results demonstrate significant racial-ethnic differences in both, opioid prescriptions and administration in the ED.

ED visits related to dental problems have garnered attention recently, due to significantly increasing trends over time [25,26,31]. Toothache is often associated with drug-seeking behavior [32–34], leading us to hypothesize that it would be similar to other non-definitive conditions and demonstrate racial-ethnic disparities in opioid prescription. A previous study also reported racial-ethnic differences in patients receiving opioids during dental ED visits, but were not significant after adjusting for covariates [35]. Again, this study did not distinguish between opioid prescription and administration. Our results indicate that there were no racial-ethnic disparities in either mode of opioid dispensing for toothache ED visits. Moreover, a large proportion (57%) of toothache ED visits resulted in prescription for opioids, resembling, in this aspect, definitive conditions. It is possible that emergency physicians are able to verify a dental disease diagnoses by examining the mouth and seeing an obvious dental pathology like a carious lesion or swelling. In that case, toothache ED visits would be treated similar to definitive conditions like fractures and kidney stones, hence limiting the subjectivity in physicians’ decision to prescribe opioids.

Many studies have suggested that drug-seeking behavior is associated with repeated ED visits [36], however, the impact of repeated past ED visits on opioid prescription has not been examined. We found that among ED visits for long-bone fracture, patients who visited the ED in the past year had lower odds of receiving opioid prescription and administration. In contrast, among ED visits for abdominal pain, those who had visited the ED more than 3 times in the past year had 50% greater odds of receiving opioid at the ED, possibly because frequent ED visitors for chronic conditions also tend to have other comorbidities, necessitating opioid administration [37–39].

As the concern for prescription drug abuse heightens [1,6,40], it is important to examine the indirect implications of efforts aimed to address this problem. Healthcare providers carry inherent human biases, which can impact their prescription practices, especially in situations that do not lend themselves well to objective decisions. Racial-ethnic minority patients, especially non-Hispanic blacks presenting with vague conditions often associated with drug-seeking behavior, may be more likely to be judged as ‘a drug-seeker’ relative to a non-Hispanic white patient, presenting with similar pain-related complains [10,12]. This is especially concerning in the light of a recent study that found that prevalence of prescription opioid abuse and addiction is lower among Hispanics and non-Hispanic Blacks, compared to non-Hispanic whites [41].

There is some evidence that patients visiting the ED on a weekend are more likely to receive opioids due to the implicit inability to visit the primary healthcare provider [36,42]. We found that ED visits on the weekends for toothache and back pain had 50% and 20% greater odds of receiving opioid prescription. It is possible that ED physicians provide opioids for pain relief over the weekend, after which patients can be expected to access further care. Compared to those with private insurance, Medicaid enrollees and the uninsured visiting ED for non-definitive conditions had lower odds of opioid being administered, likely indicating provider biases similar to race-ethnicity.

Our study has several limitations. First, each condition included in the analyses could have a variable clinical presentation, some of which was accounted by pain severity. However, other clinical indicators may provide cues to the physicians regarding the severity of the condition, hence impacting their decision to prescribe opioids. We were not able to account for such variation in clinical presentation. Also, a non-definitive reason for visit such as abdominal pain could be associated with a more definitive diagnosis, such as pancreatitis. As we did not account for such variation within each condition, we may be underestimating the disparities due to misclassification. Another factor which could affect physician’s decision to prescribe opioids is patient-initiated demand, allergies or reported adverse reactions to certain analgesics [21,36]. Given the limitations of secondary data, we were unable to assess the effect of such factors on receipt of opioids. Finally, both prescription drug abuse and under-treatment of pain in the ED are issues of concern, and several initiatives have been proposed and implemented in parts of the country to address them. We could not account for these regional variations and their overall impact on opioid prescriptions, and differential impact among racial-ethnic groups.

Despite these limitations, our study shows that there are significant racial-ethnic disparities in opioid prescription and administration in ED visits for non-definitive conditions. These disparities may reflect inherent biases that health care providers hold unknowingly, leading to differential treatment of patients based on their race. A study examining physician’s decision to prescribe opioids before and after using the drug monitoring system data found that physician decision changed in about 10% of the cases. More people received opioid prescriptions after physicians used the drug monitoring data compared to when physicians relied only on their clinical impression [21,42]. It implies that physicians tend to err on the side of caution when prescribing opioids, and our findings demonstrate that physicians are conservative more often than not, when the patient is non-Hispanic black relative to non-Hispanic white. Such biases could have serious implications by widening existing disparities, especially since racial-ethnic minorities already experience greater barriers to accessing health care [43].

On the other hand, opioid prescriptions are associated with the risk of initiating long-term addiction and abuse [44]. Recent evidence shows that non-Hispanic whites are disproportionately affected by the prescription opioid epidemic, with age-adjusted death rates more than thrice that among non-Hispanic blacks [45]. In light of this, our findings raises a perplexing question as to whether it is non-Hispanic blacks who are being under-prescribed, or is it non-Hispanic whites who are being over-prescribed. Paradoxically, then, while non-Hispanic blacks do not benefit from bias, they might be inadvertently benefitting by receiving fewer opioid medications and prescriptions [45]. Nonetheless, the access to pain management decisions should be made without regard to race and ethnicity [46]. Future interventions could include sensitization of health care providers to their inherent biases [47], hence enabling them to consciously avoid these biases from defining their practice behavior.

## Conclusions

Significant racial-ethnic disparities exist in opioid prescription and administration at ED visits for non-definitive conditions like back pain and abdominal pain, but not for other conditions like toothache, kidney stones and long-bone fracture, partially confirming our hypotheses. Such differential prescription of opioids by race-ethnicity could contribute to widening of existing disparities in health, and possibly explain the enormous burden of opioid epidemic among non-Hispanic whites to a certain extent.
